# Circulating DNA‐Based Sequencing Guided Anlotinib Therapy in Non‐Small Cell Lung Cancer

**DOI:** 10.1002/advs.201900721

**Published:** 2019-08-19

**Authors:** Jun Lu, Hua Zhong, Jun Wu, Tianqing Chu, Lele Zhang, Hua Li, Qiming Wang, Rong Li, Yizhuo Zhao, Aiqin Gu, Huimin Wang, Chunlei Shi, Liwen Xiong, Xueyan Zhang, Wei Zhang, Yuqing Lou, Bo Yan, Yu Dong, Yanwei Zhang, Baolan Li, Li Zhang, Xiaodong Zhao, Kai Li, Baohui Han

**Affiliations:** ^1^ Department of Pulmonary Medicine Shanghai Chest Hospital Shanghai Jiao Tong University Shanghai 200030 China; ^2^ School of Life Science East China Normal University Shanghai 200241 China; ^3^ Bio‐ID Center School of Biomedical Engineering Shanghai Jiao Tong University Shanghai 200240 China; ^4^ Department of Internal Medicine Henan Province Tumor Hospital Zhengzhou University Henan 450008 China; ^5^ General Department Beijing Chest Hospital Capital Medical University Beijing 101149 China; ^6^ Department of Respiratory Medicine Peking Union Medical College Hospital Chinese Academy of Medical Sciences & Peking Union Medical College Beijing 100032 China; ^7^ Shanghai Center for Systems Biomedicine Shanghai Jiao Tong University Shanghai 200240 China; ^8^ Department of Thoracic Oncology National Clinical Research Center for Cancer Tianjin Medical University Cancer Institute and Hospital Tianjin 200030 China

**Keywords:** anlotinib, liquid biopsies, non‐small cell lung cancer (NSCLC), stratification, tumor mutation index

## Abstract

Anlotinib is a multitargeted antiangiogenic drug, and its clinical predictor for responsive non‐small cell lung cancer (NSCLC) patients is still elusive. Here, tumor‐specific target capture is used to profile the circulating DNA of ALTER0303 (evaluating NSCLC clinical antitumor efficacy through anlotinib therapy) study participants. The results indicate that patients receiving no benefit can be mainly excluded via analysis of *ARID1A* and *BRCA2* genetic profiling. For patients with no durable benefit and durable clinical benefit patients, three predictors: germline and somatic mutation burden (G+S MB), nonsynonymous and synonymous mutation burden (N+S MB), and unfavorable mutation score of circulating DNA profiling are identified. Through integrating the advantages and disadvantages of three independent predictors, the tumor mutation index (TMI) is established as a prediction model and the patients who are very likely to benefit more from anlotinib therapy are identified. Furthermore, the *IDH1* ^exon 4^ mutation is identified as an unfavorable factor for anlotinib therapy under TMI‐based stratification, and the TMI plus *IDH1* ^exon 4^ mutation status potentially predicts response to anlotinib. Collectively, this study provides a circulating DNA sequencing–based stratification method for identifying anlotinib responders via a noninvasive approach, and thus potentially improves the clinical outcome of NSCLC patients receiving third‐line therapy.

## 1 Introduction

Lung cancer is the leading cause of cancer‐related death worldwide, with non‐small cell lung cancer (NSCLC) accounting for ≈85% of cases.^1^, ^2^ Effective third‐line therapy for metastatic NSCLC is still scarce. Multitargeted antiangiogenic drugs have gradually become an important option in third‐line NSCLC therapy.^3^, ^4^ However, effective clinical stratification of response to these drugs is still elusive, although the NSCLC patients have been benefitted from multitargeted antiangiogenic drug therapy.^5^ Therefore, screening of predictors of response to antiangiogenic drugs is an urgent need and will contribute to the improvement of clinical outcomes.

Following the approval of the first antiangiogenic agent bevacizumab for NSCLC therapy in 2006, intensive efforts were performed to screen for predictors of response to improve the clinical outcome of antiangiogenic drugs.^6^ Unfortunately, to date, no predictor has yet been validated for response to antiangiogenic drugs in NSCLC, although some predictors showed potential including neuropilin‐1 and VEGFR‐1 expression, short VEGF‐A isoform levels, and genetic alterations in VEGF‐A or VEGFR, among others.^6^, ^7^

Anlotinib is an effective multitargeted antiangiogenic drug used in third‐line therapy for refractory advanced NSCLC therapy in China.^8^, ^9^, ^10^ The ALTER0303 study has revealed that anlotinib significantly prolongs median progression‐free survival (PFS) (anlotinib: 5.37 months vs placebo: 1.40 months; HR: 0.25) and median overall survival (OS) (anlotinib: 9.63 months vs placebo: 6.30 months; HR: 0.68) with the objective response rate (ORR) of 9.18% and the disease control rate (DCR) of 80.95%.^11^ Our study and other recent studies have introduced some predictors for the stratification of patients on the multitargeted antiangiogenic drug anlotinib.^12^, ^13^, ^14^, ^15^ However, our understanding of the effect of the complex architecture of angiogenic signaling (especially regarding the signaling pathways underlying multitargeted antiangiogenic drugs) on predictor screening is possibly incomplete.^7^

Next generation sequencing (NGS) characterizes alterations in the chromatin genome, and has been used to screen predictors [tumor mutation burden (TMB) and microsatellite instability (MSI)] of response to checkpoint inhibitors.^16^, ^17^, ^18^ Furthermore, other genomic signatures (including gene mutation pattern,^19^, ^20^, ^21^ clone and subclone numbers,^22^ and genome evolution,^22^, ^23^) have been correlated to antitumor drugs; these findings provided potential for identifying predictors of response to anlotinib via genetic profiling. The potential for the plasma cell free DNA and circulating tumor DNA (cfDNA and ctDNA) mutational landscape to guide anlotinib therapeutic strategies has yet to be defined. Here, we performed ultradeep targeted sequencing of the plasma circulating DNA of the patients from the ALTER0303 study with the aim of identifying an effective predictor for use in anlotinib stratification of NSCLC patients receiving third‐line therapy.

## 2 Results

### 2.1 Plasma Circulating DNA Detection in NSCLC

The ALTER0303 study evaluated the antitumor efficacy of anlotinib for refractory advanced NSCLC as third‐line therapy. A targeted‐capture assay panel (168 cancer genes),^24^, ^25^, ^26^ was used to collect plasma circulating DNA (cfDNA and ctDNA) mutational information. Analytical validation of the targeted NGS platform has demonstrated sensitivity above 96% for detecting single nucleotide variants (SNVs).^25^ Massive germline and somatic mutations existed in all circulating DNA samples [111 patients at baseline (BL) and 42 patients at progression of disease (PD)]. Among the 111 advanced NSCLC patients, 35 patients with qualified paired cancer tissue samples, and showed a remarkably lower mutation burden in situ at BL (Figures S1 and S2, Supporting Information). ctDNA derived from heterogeneous cancer tissue or metastasis may account for this phenomenon.^23^ Therefore, an in‐depth understanding of the genetic profile of circulating DNA will potentially contribute to the stratification of clinical response to anlotinib [including no benefit (NB, PFS: ≤45 days), no durable benefit (NDB, PFS: 45 days < PD ≤ 130 days), and durable clinical benefit (DCB, PFS: >130 days)].

### 2.2 *ARID1A* and *BRCA2* Genetic Profiling Excludes Anlotinib NB Patients

**Figure 1:**
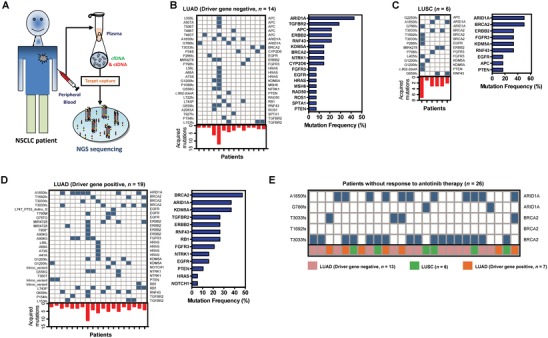
Acquired mutations in *ARID1A* and *BRCA2* are linked to anlotinib resistance in non‐small cell lung cancer via genetic alteration profiling of circulating DNA. A) Peripheral blood was collected at BL and PD, and then targeted capture–based NGS was performed to call nonsynonymous mutations/deletion mutations/insertion mutations from circulating DNA. B) Acquired mutations and corresponding genes were scattered in the 14 LUAD patients (driver gene negative). The histogram represents the acquired mutation numbers for each patient. The mutation frequency of the 17 acquired mutated genes in the 14 driver gene negative LUAD patients was also shown. C,D) Analysis of acquired mutations was performed in LUSC patients and LUAD patients (driver gene positive). E) *ARID1A* and *BRCA2* mutational analysis in NB patients at BL.

To exclude the NB patients, we first assessed the clinicopathological differences between NB patients and DCB patients, and then compared the mutational landscape from 42 DCB patients with qualified plasma circulating DNA samples. Our results indicated that alterations in mutation burden (BL vs PD) could not determine anlotinib response, but the specific acquired mutations or numbers of metastases were correlated with the response of those NB patients (**Figure** **1**; Table S1 and Figure S3, Supporting Information). Among the 42 anlotinib DCB patients, 3 patients did not acquire high‐effect nonsynonymous mutations after anlotinib therapy. Further subgroup analysis indicated 14 patients with lung adenocarcinoma (LUAD) (driver gene negative) and 19 patients with LUAD (driver gene positive), and 6 patients with lung squamous cell carcinoma (LUSC), and found different acquired mutation patterns in the different subgroups (Figure 1B–D).

We hypothesized that the acquired mutations are correlated with acquired anlotinib resistance, and then analyzed the acquired mutations in the three subgroups independently. Our results indicated that 14 LUAD patients (driver gene negative) acquired mutations (range: 1–13 mutations) in 17 cancer genes after resistance to anlotinib occurred (Figure 1B, left). Mutation frequencies of *ARID1A* (43%) and *TGFBR2* (29%) were the two most frequently mutated genes (Figure 1B, right). Some differences were found after evaluating acquired genetic alterations in LUSC patients (*n* = 6). Of the acquired mutations (range: 1–7 mutations) in 9 cancer genes, the mutation frequencies of *ARID1A* (50%) and *BRCA2* (50%) were the highest (Figure 1C). The driver gene positive LUAD patients acquired mutations (range: 1–11 mutations) in 13 genes, and the mutation frequencies of *BRCA2* (47%) and *ARID1A* (37%) were the highest (Figure 1D). In total, the acquired mutations in *ARID1A* (41%) and *BRCA2* (36%) mutations had highest mutation frequencies in all 39 patients with acquired mutations. These results indicated that acquired mutations in *ARID1A* and *BRCA2* may be associated with acquired anlotinib resistance.

We then investigated whether the mutations in *ARID1A* and *BRCA2* were initially involved in the resistance to anlotinib in NSCLC. Five point mutations in *ARID1A* and *BRCA2* were examined in all 26 anlotinib NB patients (13 driver gene negative LUAD, 6 LUSC, and 7 driver gene positive LUAD) at BL, and the results indicated that 92% (24/26) of NB patients harbored *ARID1A* (A1850fs, chr 1: 26 779 439:TG/T and G766fs: chr 1: 26 762 190:TC/T) and *BRCA2* (T3033fs: chr 13: 32 379 885:CA/C and chr 13: 32 379 885:C/CA) mutations, suggesting these acquired point mutations are potentially associated with anlotinib resistance (Figure 1E). Therefore, vast majority of NB patients can be excluded via *ARID1A* and *BRCA2* profiling at BL before anlotinib therapy.

### 2.3 Germline and Somatic Mutation Burden (G+S MB), and Nonsynonymous and Synonymous Mutation Burden (N+S MB) for Anlotinib Responsive Stratification

**Figure 2:**
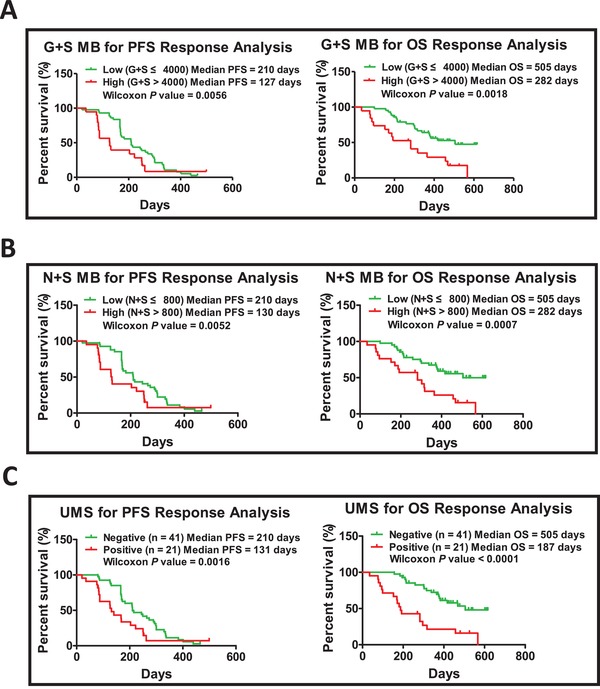
Mutational burden used as a predictor for anlotinib response analysis of PFS and OS in the discovery cohort. A) Kaplan–Meier plots of PFS and OS in NSCLC patients receiving anlotinib, when the predictor G+S MB cutoff was set at 4000. Patients with plasma circulating DNA harboring lower G+S MB (*n* = 43) compared to those harboring higher G+S MB (*n* = 19) (PFS: 210 days vs 127 days, Wilcoxon *P* = 0.0056; OS: 505 days vs 282 days, Wilcoxon *P* = 0.0018). B) Plasma circulating DNA with lower N+S MB (*n* = 41) compared to those with higher N+S MB (*n* = 21) (PFS: 210 days vs 130 days, Wilcoxon *P* = 0.0052; OS: 505 days vs 282 days, Wilcoxon *P* = 0.0007). C) PFS and OS in patients with a negative UMS (*n* = 41) compared to those with a positive UMS (*n* = 21) (PFS: 210 days vs 131 days, Wilcoxon *P* = 0.0016; OS: 505 days vs 187 days, Wilcoxon *P* < 0.0001).

Among the 111 NSCLC patients, excluding the 26 NB patients, the stratification based on response of remaining 85 patients (including DCB and NDB) was still elusive. Mutation burden has been used for checkpoint inhibitors response stratification in both tissue and plasma.^17^, ^18^ Here, we assumed that the mutation burden potentially served as a predictor that could be used for anlotinib response stratification, and allocated 62 patients as the discovery cohort, and 23 patients as the validation cohort (Figure S4A, Supporting Information). Interestingly, we found that patients with lower G+S MB (G+S < 4000) were more sensitive to anlotinib therapy than those with a higher burden (PFS: 210 days vs 127 days, Wilcoxon *P* value = 0.0056; OS: 505 days vs 282 days, Wilcoxon *P* value = 0.0018) (**Figure** **2**A). Under this stratification, patients with low G+S MB presented similar mutation burden between plasma and tissue, while patients with high G+S MB presented significant difference between plasma and tissue (Figure S2B,C, Supporting Information). Further analysis indicated that G+S MB could screen anlotinib responders from NDB and DCB patients credibly (Figure S5A–D, Supporting Information). Composition analysis demonstrated a similar pattern with clinical characteristics in the discovery cohort, except the nonsmokers (Figure S6A and Table S2, Supporting Information). Response analyses on subgroups suggest the predictor of G+S MB can remarkably distinguish anlotinib responders from the nonresponders in those subgroups of male, smoker, and negative driver gene (Table S3, Supporting Information). These results demonstrated that mutation burden could potentially be used as a predictor for anlotinib response stratification in NDB and DCB patients.

To screen out preferable predictors used in anlotinib response stratification, we next asked whether N+S MB could also serve as predictor for anlotinib stratification. To address this issue, we filtered out the germline mutations and the low influence mutations, yielding the N+S MB. Similar to the predictor of G+S MB, the patients harboring a lower N+S MB were sensitive to anlotinib, while those harboring a higher N+S MB were poorly sensitive to anlotinib (PFS: 210 days vs 130 days, Wilcoxon *P* value = 0.0052; OS: 505 days vs 282 days, Wilcoxon *P* value = 0.0007) (Figure 2B). Distribution pattern of N+S MB between tissue samples and plasma samples is also similar to the characteristics of G+S MB (Figure S2D–F, Supporting Information). However, characteristics of the predictor of N+S MB are different, and the predictive value of N+S MB is better than G+S MB on the whole (Table S1 and Figures S5A–F and 6A,B, Supporting Information).

### 2.4 Determinants of Unfavorable Mutation Score (UMS) and Its Application to Anlotinib Stratification

For further stratification of these NDB and DCB patients, we assumed that specific mutations are associated with anlotinib response, and hence sought to screen for potential unfavorable mutations (Figures S4A and S7, Supporting Information). The correlation analysis of anlotinib response and mutation in the discovery cohort identified 120 unfavorable mutations (Wilcoxon *P* value < 0.01). These unfavorable mutations were distributed in 58 cancer genes, and each gene contained 1–14 mutations (Figure S7A, Supporting Information). The mutation frequency of each gene varied from 8% to 26% (Figure S7B, Supporting Information). The UMS was derived from patients harboring unfavorable mutation numbers, and ranged from 0 to 96 (Figure S5B, Supporting Information). The UMS identified in plasma circulating DNA was also remarkably higher than that in tissue DNA (Figure S2G,H).

Similar to the predictors of G+S MB and N+S MB, the patients who had a lower UMS received more benefit from anlotinib therapy than those with a higher UMS (Figure S5A, Supporting Information). Further analysis demonstrated that NSCLC patients with a negative UMS benefited more from anlotinib therapy (Figure 2C; Figure S5G,H, Supporting Information). However, the patients characterized as nonsmoker and with >3 metastases accounted for the majority of the UMS positive patients (Figure S6C, Supporting Information). As a predictor for anlotinib response stratification, UMS was suitable for 60% subgroups (except for the subgroups of female, nonsmokers, those with a positive driver gene, and those with >3 metastases) (Table S3, Supporting Information). Overall, UMS as a predictor showed better predictive value than the predictors of G+S MB and N+S MB, both in the total discovery cohort and their subgroups.

### 2.5 Establishment of the Tumor Mutation Index (TMI) and Its Use in Anlotinib Response Stratification

**Figure 3:**
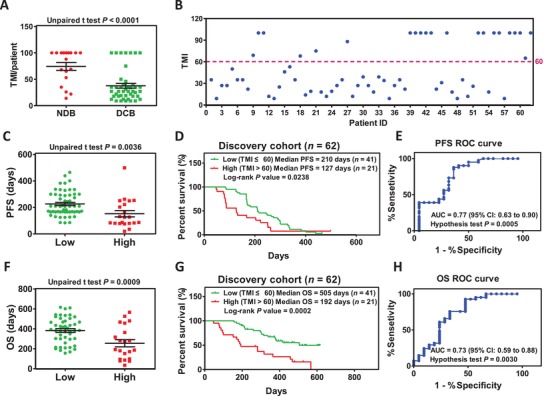
TMI as a predictor of anlotinib response in the discovery cohort. A) TMI in patients with DCB (*n* = 44) compared to NDB (*n* = 18) (*P* < 0.0001). B) Distribution of TMI for each patient. Cutoff = 60. C,F) PFS analysis between the patients with lower TMI (*n* = 41) and those with higher TMI (*n* = 21) (unpaired *t* test *P* < 0.0036). A similar analysis was performed for OS (unpaired *t* test *P* = 0.0009). D,G) Kaplan–Meier curve analysis for predicting anlotinib response regarding PFS (low TMI: 210 days vs high TMI: 127 days, log‐rank *P* = 0.0008). Similar analysis was performed for OS (low TMI: 505 days vs high TMI: 192 days, log‐rank *P* = 0.0002). E,H) ROC curves for the correlation of TMI with anlotinib response. AUC of PFS response prediction is 0.77 (95% CI 0.63 to 0.90, null hypothesis test *P* = 0.0005) and AUC of OS response prediction is 0.73 (95% CI 0.59 to 0.88, null hypothesis test *P* = 0.0030). Cutoff = 60 determined by the Ward method. In panels (A), (C), and (F), median and interquartile ranges of total TMI are shown, with individual values for each patient shown with dots.

**Figure 4:**
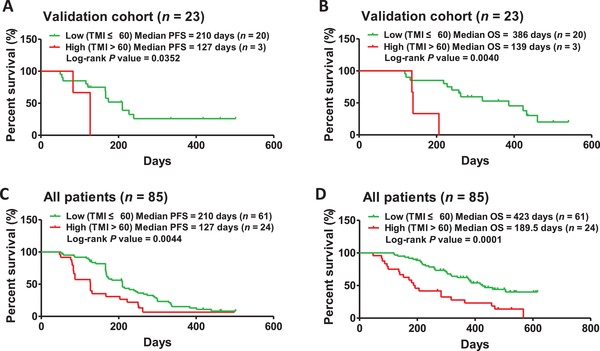
TMI used for stratifying anlotinib responders in the validation cohort and all patients. A) Stratification analysis based on PFS between the patients with lower TMI (*n* = 20) and those patients with higher TMI (*n* = 3) in validation cohort (210 days vs 127 days, log‐rank *P* = 0.0352). B) OS in the patients with lower TMI (*n* = 20) compared to those with higher TMI (*n* = 3) in patients in validation cohort (386 days vs 139 days, log‐rank *P* = 0.0040). C,D) Responsive stratification using the predictor‐TMI was performed in all 85 patients (PFS: 210 days vs 127 days, log‐rank *P* = 0.0044; OS: 423 days vs 189.5 days, log‐rank *P* = 0.0001).

Interestingly, we found different predictive values for subgroups when the three predictors were used for anlotinib stratification respectively (Table S3, Supporting Information). Therefore, we hypothesized a predictive model‐TMI that could integrate the merits and defects of the predictors G+S MB, N+S MB, and UMS (Figure S4, Supporting Information). After calculation of the TMI score of each patient, the TMI score of NDB patients was found to be significantly higher than that of DCB patients (*P* < 0.0001) (**Figure** **3**A). Vice versa, patients harboring low TMI scores were more responsive to anlotinib therapy than those with higher TMI scores (Figure 3B,C,F). Kaplan‐Meier curve analysis and receiver operator characteristic (ROC) curve analysis indicated that TMI as a predictor is preferable to G+S MB, N+S MB, and UMS (Figure 3D,E,G,H). Except the subgroups of nonsmoker and >3 metastases, predictor of TMI is suitable for all subgroups in discovery cohort (Table S3, Supporting Information). As to the subgroups of nonsmokers and >3 metastases, imbalanced composition maybe contributes majority cause (Figure S6D, Supporting Information). TMI as a predictor performed better regarding the null hypothesis test *P* value and responsive stratification of subgroups. Validation analysis of TMI as a predictor independently in 23 patients and totally in 85 patients suggested a preferable distinction between anlotinib responders and anlotinib lower responders (**Figure** **4**).

### 2.6 TMI Combination with *IDH1* ^exon 4^ Mutation Status Predicts Response to Anlotinib

**Figure 5:**
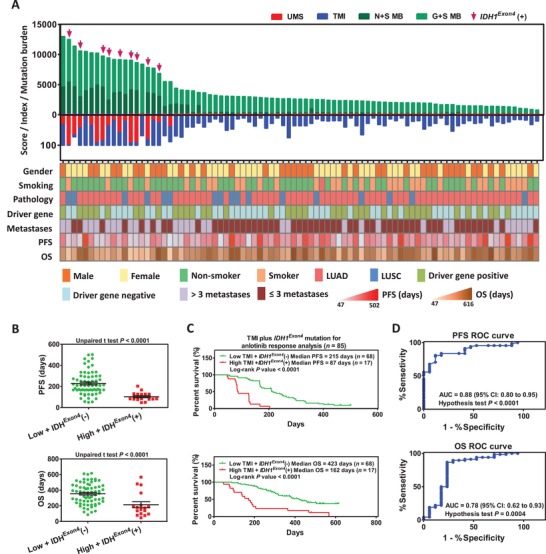
TMI plus *IDH1* ^exon 4^ mutation status used as predictor for anlotinib responsive stratification. A) Integrated mutational landscapes and clinical factors were correlated with anlotinib response in NDB and DCB patients. B) PFS in the patients with a low TMI plus *IDH1−* (*n* = 68) compared with PFS in those with a high TMI plus *IDH1+* (*n* = 17) (unpaired *t* test *P* < 0.0001). Similar comparison was performed on OS (unpaired *t* test *P* < 0.0001). Median and interquartile ranges of total mutations are shown, with individual values for each patient shown with dots. C) Kaplan–Meier curves for anlotinib response analysis via the predictor of TMI plus *IDH1* ^exon 4^ mutation status (PFS: 215 days vs 87 days, log‐rank *P* < 0.0001; OS: 423 days vs 162 days, log‐rank *P* < 0.0001). D) ROC curves for the correlation of TMI plus *IDH1*+ with anlotinib response. AUC of PFS response prediction was 0.88 (95% CI 0.80 to 0.95, null hypothesis test *P* < 0.0001) and AUC of OS response prediction was 0.78 (95% CI 0.62 to 0.93, null hypothesis test *P* = 0.0005).

Although the TMI score showed satisfactory stratification for predicting response to anlotinib, we still found that some patients with full TMI scores had a good response to anlotinib therapy (Figure 3A). Therefore, understanding the underlying genetic differences of these patients will contribute to the improvement of TMI‐based stratification. Genetic analysis revealed that 6 patients (with a higher response to anlotinib) did not have the *IDH1* ^exon 4^ mutation, and 9 of the remaining 10 patients (with a lower response to anlotinib) had the *IDH1* ^exon 4^ mutation. These results suggested that the patients (full TMI score) without the *IDH1* ^exon 4^ mutation may be suitable for anlotinib therapy (Figure S8, Supporting Information). Therefore, we integrated all factors (including the UMS, TMI, N+S MB, G+S MB, *IDH1* ^exon 4^, gender, smoking, pathology, driver gene, metastases, PFS, and OS) and found that predictor of TMI in combination with *IDH1* ^exon 4^ mutation status could effectively exclude the lower‐responders from NDB and DCB patients (**Figure** **5**). The results indicated that patients with high TMI scores plus *IDH1* ^exon 4^ mutation positivity received a reduced lower benefit from anlotinib therapy compared to the other patients (low TMI plus *IDH1* ^exon 4^ mutation negative patients in those full score of TMI) (PFS: log‐rank *P* value < 0.0001, AUC = 88%; OS: log‐rank *P* value < 0.0001, AUC = 78%) (Figure 5B–D).

## 3 Discussion

The tumor mutational landscape correlates with response to checkpoint inhibitors in NSCLC therapy,^17^, ^18^, ^27^ but the predictive values for multitargeted antiangiogenic drugs have not yet been reported. Here, we provide a response stratification approach based on the different mutational landscapes of patients from the ALTER0303 study by characterizing SNVs of circulating DNA; this revealed different anlotinib response characteristics. By integration analysis, we found that circulating DNA‐based sequencing at BL could effectively predict the anlotinib response.

These observations may have an important clinical implication for guiding third‐line anlotinib therapy in NSCLC. The majority of NSCLC patients rejected tumor tissue biopsy in situ before receiving third‐ or over third‐line therapies.^26^ Even if patients accept tissue biopsy, the limited information precludes the establishment of a comprehensive mutational landscape due to heterogeneity.^22^ A similar issue occurs during the biopsy of surface lymph node metastasis. ctDNA enters the plasma from different regions of in situ tumor or different metastases in advanced NSCLC patients after 2rd‐ or over 2rd‐line therapies, possibly providing more comprehensive mutational information.^23^ Most of these patients experienced long‐term tumor evolution and carried more clone SNVs or subclone SNVs. This process has been demonstrated in multiregion tissue sequencing.^22^ Through sequencing of cfDNA and ctDNA, our study found that some NSCLC patients were harboring more mutations in plasma than in tissue, which confirmed the limitations of sequencing single tumor region. Once the tumor burden reaches 10 cm^3^, it results in a mean clonal plasma variant allele frequency (VAF) of 0.1%,^23^ suggesting the characterizing mutations of cfDNA and ctDNA can effectively guide late‐stage clinical therapies.

The use of genetic alterations^28^ (such as gene amplification, point mutation, gene overexpression, and chromosomal translocation) and genomic landscape alterations^16^, ^18^, ^22^, ^23^ (such as gene mutation pattern, MSI, clone and subclone number, and genome evolution) in predicting the response to antitumor drugs has potential applications in clinical practice. However, there is no suitable predictor that can be used to distinguish responders to antiangiogenic drugs, especially on multitargeted antiangiogenic drugs.^29^ Angiogenesis‐related receptors (such as VEGFR, PDGFR, and FGFR) can be remarkably inhibited via exposing exposure to multitargeted antiangiogenic drugs, but no confirmed clinical evidence has validated the correlation between expression of angiogenesis‐related factors and clinical outcome due to the complex architecture of angiogenic signaling.^6^, ^8^, ^9^, ^11^ Our results found that NSCLC patients are initially resistant to multitargeted antiangiogenic anlotinib, once they acquire *ARID1A* and *BRCA2* mutations. Mutational burden correlates with the use of checkpoint inhibitors in predicting response,^18^, ^30^ which provides a new perspective for identifying predictors of anlotinib response. Here, we found that plasma G+S MB was a predictor that could significantly distinguish anlotinib responders, especially in some subgroups with a PFS of 210 days or more than 240 days in responders. While examining the predictor of N+S MB and UMS for predicting anlotinib response, we found that these predictors have different predictive values in different subgroups, although a similar median PFS and OS were observed in the discovery cohort, suggesting inner flaws affected the stratification of response to multitargeted antiangiogenic drugs using unique predictor. Namely response to anlotinib is also associated with the basic attributes of a patient (such as gender, smoking history, pathology type, driver gene status, and number of metastases). Therefore, here we performed preliminary stratifications of multitargeted antiangiogenic drug response based on a multifactors, integrated TMI.

A predictor derived from plasma is expected to span a range of disciplines in the future.^31^ Comprehensive analysis of the mutational information existing in plasma cfDNA and ctDNA will improve clinical outcomes, but this is still not well‐understood at present. A previous study indicated that tumors harboring more mutations produce extensive neoantigens, which enhances efficacy of checkpoint inhibitors.^18^ Unlike the findings regarding checkpoint inhibitors, the present findings indicated that multitargeted antiangiogenic therapy using anlotinib brings neglected survival benefit to the NSCLC patients harboring more mutations (especially with a high TMI), but provides significant survival benefit to those harboring fewer mutations (especially those with a low TMI). A single predictor reflects most of events, but can be improved via other factors.^16^, ^18^, ^30^, ^32^ A previous study described NSCLC patients harboring STK11 mutation who had primary resistance to checkpoint inhibitors, although they had a high TMB.^33^ A similar phenomenon also exists in fist‐generation TKI therapy for those patients harboring concomitant mutations.^34^, ^35^ While our results showed that TMI could predict anlotinib response effectively, further analysis indicated that not all TMI full scored patients had a poor response to anlotinib. If those patients did not have the *IDH1* ^exon 4^ mutation, they received commendable benefit from anlotinib therapy. Finally, this study suggested an important beneficial subgroup via profiling of plasma circulating DNA. A prospective trial will continue to evaluate the validity of the circulating DNA‐based sequencing for NSCLC patients treated with anlotinib.

Whether the mutational landscape derived from plasma circulating DNA sequencing for anlotinib stratification can be validated in a prospective trial warrants further attention. In addition to ongoing efforts to discuss circulating DNA‐based sequencing for anlotinib stratification, there is a need to develop a greater understanding of proteomics and exosome omics, since the altered proteins possibly limit the efficacy of multitargeted antiangiogenic drugs. In the analysis presented here, we provide mutational panoramic predictors based on a circulating DNA sequencing platform for guiding the clinical use of anlotinib for NSCLC as a third‐line therapy.

## 4 Experimental Section

*Patients and Samples*: In total, 440 advanced NSCLC patients were enrolled in the ALTER‐0303 study (https://clinicaltrials.gov/NCT02388919). Of the 440 patients, 126 patients (placebo: 15 patients; anlotinib: 111 patients) with qualified samples (including white blood cell (WBC), blood, and tissue) were analyzed in the present study (Figure S1, Supporting Information). All refractory advanced NSCLC patients were enrolled in Shanghai Chest Hospital, Chinese Academy of Medical Sciences Cancer Hospital, Peking Union Medical College Hospital, etc. All patients had received at least two lines of targeted therapy or chemotherapy, and had failed prior therapies. The patients were orally administered with anlotinib as a third‐line therapy or over third‐line therapy with a dosage of 12 mg day^−1^ for two consecutive weeks that was then discontinued for one week. If PD or intolerable toxicity occurred, anlotinib therapy was terminated immediately. Multicenter plasma and tumor collection was performed as previously described.^9^, ^11^ Clinical information of each patient is shown in Tables S4 and S5 in the Supporting Information. Informed consent was obtained from all subjects following the ALTER‐0303 study.

*Pathological Type and Staging*: EGFR driver gene mutations were detected in tissue DNA by ADx‐ARMS method, and ALK fusion or ROS1 rearrangement were detected in tissue RNA via RT‐qPCR method. The patient harboring any one of these positive mutations in EGFR, ALK, and ROS1 was defined as driver gene positive. Tumor volume and metastases were evaluated on the basis of CT scans by at least one radiologist. Stages for each patient were determined by at least one investigator.

*Tissue DNA Extraction and Sequencing*: A customized targeted capture assay panel (168 cancer genes, Burning Rock Dx) was used to capture target DNA.^24^, ^25^, ^26^ Briefly, DNA was extracted from tumor tissue slides according to the standard procedures. Targeted capture was performed on at least 200 ng of input DNA for each sample. After amplifying captured DNA, high‐throughput sequencing was performed to collect raw data for genomic information. Trimmomatic (version 0.36) was used to trim low quality bases of raw reads.^36^ Cleaned data were aligned to the latest human genome assembly hg38 using Burrows–Wheeler Aligner (BWA) with default parameters.^37^ Mutations were called with Varscan2 with default parameters for each sample.^38^

*Circulating DNA Extraction*: Blood samples for each patient were collected in a 10 mL K2‐EDTA tube. All plasma samples were collected within 2 h of collection by centrifugation of blood samples at 1600 × *g* for 10 min. Then, the upper plasma was transferred to 1 mL cleaned Eppendorf tubes using a pipette, and the tubes were sequentially marked. Plasma was stored at −80 °C. Up to 5 mL of plasma from each patient was available for this study (range, 3–5 mL). cfDNA and ctDNA were extracted from the entire volume of plasma using the QIAamp Circulating Nucleic Acid Kit (Qiagen). All cfDNA and ctDNA samples were eluted in 50 µL of DNA buffer (0.05 m, pH: 7.5). cfDNA and ctDNA quantification was performed by the Qubit fluorescence quantitative method (Invitrogen).

*Library Preparation*: Tumor tissue DNA (200–300 ng) or plasma cfDNA and ctDNA (10–100 ng) for each sample was used for targeted exome capture. Library preparation was performed as previously described.^24^, ^25^, ^26^ Captured DNA for each sample was end‐repaired and adaptor ligated, and then amplified for no more than 12 cycles in a thermal cycler (Applied Biosystems). Finally, the PCR products were quantified using Qubit (Thermo), and underwent paired‐end sequencing using a 2*150 model.

*Plasma SNV Calling*: Quality analysis of raw sequencing data were performed based on the authors' and other previous studies.^24^, ^25^, ^26^ The SNV calling algorithm was performed as previously reported.^23^ WBC samples were used to estimate the error parameters for calling SNVs. Germline and somatic mutations were obtained via calculating sequencing depth (≥100×) and VAF. All germline and somatic mutations were annotated, and then the genes that were not included in the scope of 168 genes were filtered. According to the methods reported in a previous study, the mutations were filtered with VAF > 20%,^23^ the mutations were deleted with low effect (MODIFIER and LOW), and finally the mutations were obtained with relatively high effect (MODERATE and HIGH). These mutations were defined as somatic mutations (synonymous mutations and nonsynonymous mutations). The synonymous mutations were filtered, and then nonsynonymous mutations were remained. The germline and somatic mutations, the somatic mutations, and the nonsynonymous mutations were sequentially obtained, for each patient.

*Acquired Mutation Analysis*: Totally 42 DCB patients were performed to compare the genetic alteration (nonsynonymous mutations with high affect) between BL and PD. Acquired mutation analysis was performed on the subgroups of driver gene (EGFR, ALK, and ROS1) negative lung adenocarcinoma patients (*n* = 14), lung squamous carcinoma patients (*n* = 6), and driver gene positive LUAD patients (*n* = 19), respectively. The types of acquired mutations, the numbers of acquired mutations, and the mutation frequency of acquired mutational genes were analyzed.

*Analysis of Acquired Mutations in NB Patients*: Totally 26 NB patients were performed with the same cfDNA and ctDNA profiling at BL. The mutations with top frequency were compared to the landscape of each NB patient. The correlation between acquired mutations and initial anlotinib resistance was discussed based on the data generated in 40 anlotinib DCB patients and 26 anlotinib NB patients.

*Ward Method for Cutoff Determination*: Survival analysis was performed to obtain significance *P* values by calculating the correlation between predictors (G+S MB, N+S MB, and UMS) and PFS/OS, and Kaplan–Meier plots were made with the R package “survival” or GraphPad Prism 5. According to mutation burden or TMI (from low to high), the *P* value of stratification was obtained sequentially. The *P* values were compared, and then the lowest *P* value set as the cutoff was selected out. This method is suitable for all PFS and OS analysis.

*Clinical Efficacy Analysis*: Objective response to anlotinib was evaluated by at least one investigator according to CT scan. Here, the patients with stable disease or partial response lasting 130 days were defined as DCB, while those patients with 45 days < PD ≤ 130 days were defined as NDB, and the patients with PD ≤ 45 days or anlotinib intolerance were defined as NB. For patients with ongoing response to anlotinib therapy, PFS was censored at the date of the most recent imaging evaluation. For the factor of alive or death, OS was censored at the date of last known contact.

*G+S MB and N+S MB for Anlotinib Response*: Kaplan–Meier curve analysis was performed to evaluate the correlation between mutation burden and anlotinib response. The cutoff *P* value was determined by the “Ward method.” Determination of ongoing response and living status was described as “clinical efficacy analysis.” The significance *P* value was obtained by comparing the median PFS or median OS between those with a high mutation burden and with a low mutation burden. The ROC curves for predicting PFS and OS were generated by the cutoff *P* value of mutation burden using GraphPad Prism. AUC (95% CI) and null hypothesis test *P* were determined by ROC.

*UMS Used for Anlotinib Response Analysis*: The mutation tables were generated with a custom Python script, in which each row indicated a specific mutation and each column indicated a sample. Each cell of the mutation table denoted the sequencing depth and VAF of the corresponding mutation. Survival analysis was performed for the samples of the discovery cohort at BL using R package “survival” for each single mutation. Patients were classified into 2 groups (positive or negative) based on whether the patient had this mutation. Each mutation was examined against the PFS to test whether this mutation could significantly reduce the PFS for the mutation‐positive group. Then the Wilcoxon *P* value was adjusted by the BH method. A total of 120 candidate mutations passed the cutoff with the adjusted *P* value. These mutations served as candidates that could significantly decrease PFS.

Finally, based on the 120 candidate mutations, a scoring system was developed to evaluate the risk of the patient. Each positive mutation shared the same weight and was scored as 1. For example, one patient would receive a score of 10 if the patient had 10 such mutations. Then, patients were grouped into 2 groups based on the scoring system, namely, the negative (no such mutation) and high‐risk (more than 1 mutation) groups. Then, Kaplan–Meier survival analysis was performed against with PFS or OS using the same method as above to test whether such a scoring system could differentiate low‐ and high‐risk patients.

*TMI Generation*: The process of generating the TMI is shown in Figure S4 in the Supporting Information. TMI is based on three different anlotinib predictors (G+S MB, N+S MB, and UMS). Distinguishing anlotinib responders and anlotinib nonresponders using above three predictors, each anlotinib responder will score 50 points as BL. According to the significance of Kaplan–Meier curve analysis and ROC curve analysis upon different predictors, the significantly different *P* values < 0.05 scored 1, *P* values < 0.01 scored 2, and *P* values < 0.001 scored 3, in Kaplan–Meier curve analysis for PFS and OS. AUC values > 0.7 scored 1, and a null hypothesis test *P* value < 0.05 scored 1, < 0.01 scored 2, and < 0.001 scored 3 in ROC curve analysis for sensitivity and specificity. A score was allocated to each subgroup according to the above standards. Each patient obtained a score based on the characters of demographic data (such as gender, smoking status, LUAD, negative driver gene, and ≤3 metastases). Under the scoring approach, each patient obtained three independent BL scores and subgroup scores based on three predictors (G+S MB, N+S MB, and UMS). The six values above were added together to obtain a total score for each patient. Homogenization was performed according to the formula TMI = 100 × (300−score)/300, and then the TMI score was obtained for each patient. The TMI was used as a predictor, the “Ward method” was performed to determine the cutoff, Kaplan–Meier curve analysis was used to test anlotinib response stratification, and ROC curve analysis was performed to evaluate the predictive value.

*Composition Analysis*: According to the demographic characteristics, all the patients were divided into 10 subgroups (male, female, smoking, non smoking, LUAD, LUSC, driver gene positive, driver gene negative, >3 metastases, and ≤3 metastases). The composition of each subgroup was distinguished by the obtained predictors, such as the proportion of men and women in G+S MB <4000, and among others.

*Anlotinib Response Analysis in Subgroups Using the Predictors of G+S MB, N+S MB, UMS, and TMI*: In the discovery cohort, anlotinib response analysis was performed on different subgroups using the above predictors. After these analyses, the *P* values of Kaplan–Meier curve analysis of the PFS/OS, the AUC values of area under ROC curve, and null hypothesis test *P* values were used for subgroup response analysis.

*Data Availability*: Clinical information and predictor scores for this cohort can be found in NCBI database. The BioSample accession address is https://www.ncbi.nlm.nih.gov/biosample, Submission ID: SUB1189225. Mutation list called by Varscan2 with default parameters appeared in GTR database. The Laboratory accession number is GTR000568272; the Submission ID is SUB5954608. These data are also shown in Tables S4–S6. Providing access to the raw sequencing reads was not possible due to the restrictions of the project supporter (Chia‐tai Tianqing Pharmaceutical Co. Ltd.). Raw sequencing data sharing was upon request to Dr. Baohui Han (xkyyhan@gmail.com, 18930858216@163.com).

*Statistical Analysis*: The Wilcoxon test was used to compare Kaplan–Meier curves during TMI generation. A log‐rank test was used to compare Kaplan–Meier curves in the validation cohort and subsequently stratify the analysis. Unpaired *t* test was used to compare the mutation burden between DCB and NDB. The ROC curve was determined by plotting the rate of DCB at various cutoff settings of predictors. That is, the proportion of all DCB patients with a mutation burden above any given cut point (sensitivity) was plotted against the proportion of the NDB patients who would also exceed the same cutoff point (1− specificity). The AUC and exact 95% confidence intervals were reported. To examine the credibility of stratification, null hypothesis test was performed to analyze the ROC curve. Statistical analyses were performed using GraphPad Prism 5. Differences were considered significant at *P < 0.05, **P < 0.01, and ***P < 0.001.

## Conflict of Interest

The authors declare no conflict of interest.

## Supporting information

Supplementary
