# Supplementary material for: Circulating DNA‐Based Sequencing Guided Anlotinib Therapy in Non‐Small Cell Lung Cancer
Source: Adv Sci (Weinh). 2019 Aug 19;6(19):1900721. doi: 10.1002/advs.201900721 (PMC6774020; doi:10.1002/advs.201900721)
Supplement: Supplementary file 1 — Supplementary [file ADVS-6-1900721-s001.pdf]

Copyright WILEY-VCH Verlag GmbH & Co. KGaA, 69469 Weinheim, Germany, 2019.

ADVANCED SCIENCE
Open Access

Supporting Information

for *Adv. Sci.,* DOI: 10.1002/advs.201900721

Circulating DNA-Based Sequencing Guided Anlotinib Therapy in Non-Small Cell Lung Cancer

Jun Lu, Hua Zhong, Jun Wu, Tianqing Chu, Lele Zhang, Hua Li, Qiming Wang, Rong Li, Yizhuo Zhao, Aiqin Gu, Huimin Wang, Chunlei Shi, Liwen Xiong, Xueyan Zhang, Wei Zhang, Yuqing Lou, Bo Yan, Yu Dong, Yanwei Zhang, Baolan Li, Li Zhang, Xiaodong Zhao, Kai Li, and Baohui Han**

Supplementary Figures

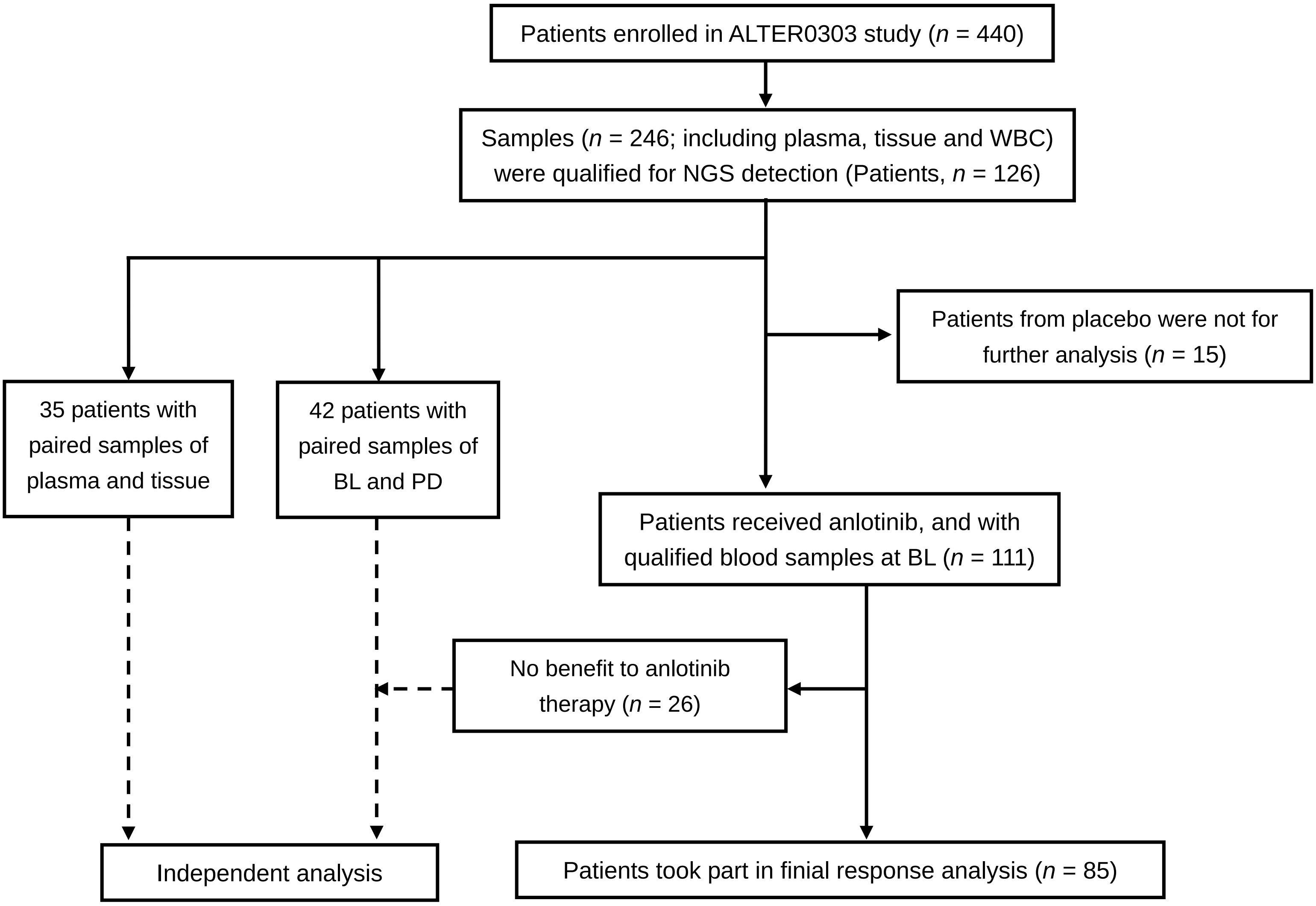

Supplementary Figure 1. Flow diagram of patient selection.

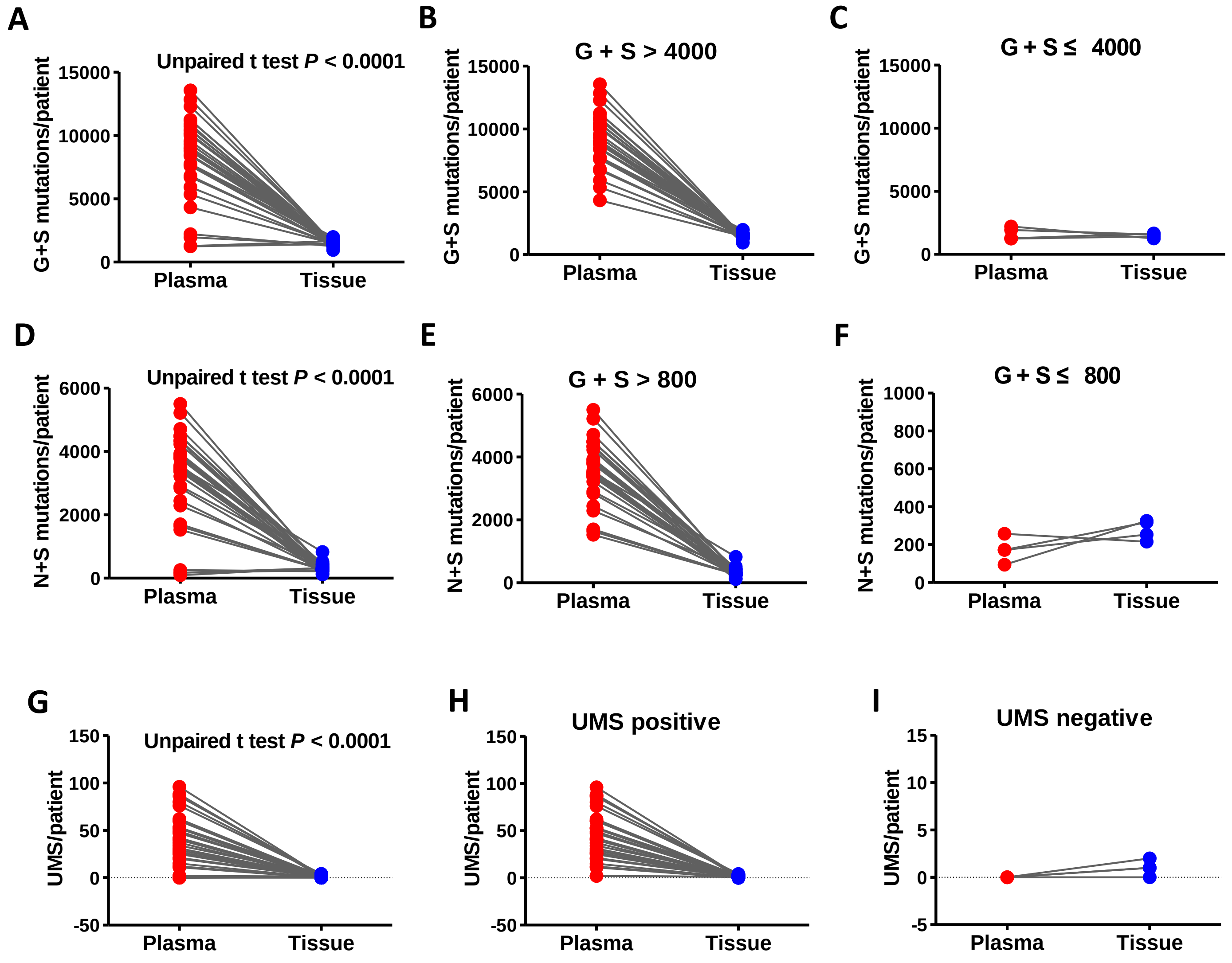

Supplementary Figure 2. Mutations from circulating DNA compared with mutations from tissue. Plasma and tumor tissue *in situ* were obtained at BL. In total, 35 patients had paired samples, and the sequencing data met the requirements. (A) Distribution of germline and somatic (G+S) mutations between plasma and tissue. (B) G+S mutation number comparison between plasma and tissue, with mutations over 4000 in circulating DNA (cfDNA and ctDNA). (C) G+S mutation comparison between plasma and tissue, with mutations under 4000 in cfDNA and ctDNA. (D-F) The germline mutations were filtered, and nonsynonymous and synonymous mutation (N+S) differences between plasma and tissue were observed. Cutoff = 800. The different patterns between plasma and tissue, such as the high N+S mutation

compared with the low N+S mutation. (G-I) Analysis of UMS derived from plasma and tissue, respectively. UMS positive in plasma compare to tissue and UMS negative in plasma compare to tissue, respectively.

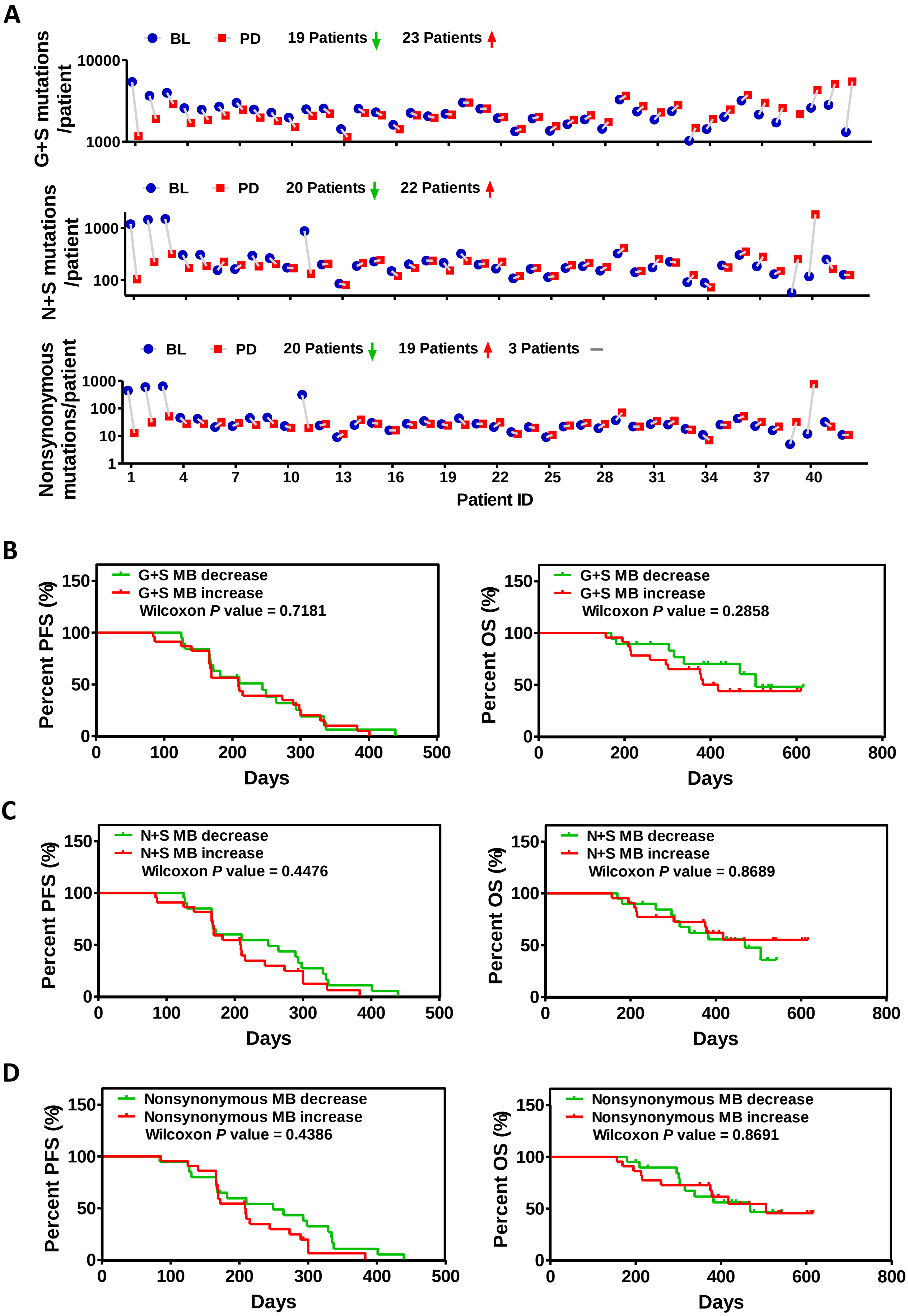
A
BL
PD
19 Patients
23 Patients
G+S mutations /patient
10000
1000
BL
PD
20 Patients
22 Patients
N+S mutations /patient
1000
100
BL
PD
20 Patients
19 Patients
3 Patients
Nonsynonymous mutations/patient
1000
100
10
1
1
4
7
10
13
16
19
22
25
28
31
34
37
40
Patient ID
B
G+S MB decrease
G+S MB increase
Wilcoxon P value = 0.7181
Percent PFS (%)
Days
G+S MB decrease
G+S MB increase
Wilcoxon P value = 0.2858
Percent OS (%)
Days
C
N+S MB decrease
N+S MB increase
Wilcoxon P value = 0.4476
Percent PFS (%)
Days
N+S MB decrease
N+S MB increase
Wilcoxon P value = 0.8689
Percent OS (%)
Days
D
Nonsynonymous MB decrease
Nonsynonymous MB increase
Wilcoxon P value = 0.4386
Percent PFS (%)
Days
Nonsynonymous MB decrease
Nonsynonymous MB increase
Wilcoxon P value = 0.8691
Percent OS (%)
Days

Supplementary Figure 3. No correlation upon mutation burden alteration (from BL to PD) and anlotinib response. (A) The alterative mutation burden from BL to PD in each patient. *n* = 42. The green arrow means mutation burden decrease, the red arrow means mutation burden increase, and the horizontal line means no mutation burden alteration. (B-D) Kaplan-Meier curves analysis for comparing the correlations between mutation burden alterations (G+S MB, N+S MB, and Nonsynonymous mutation burden) and anlotinib response, on PFS and OS respectively.

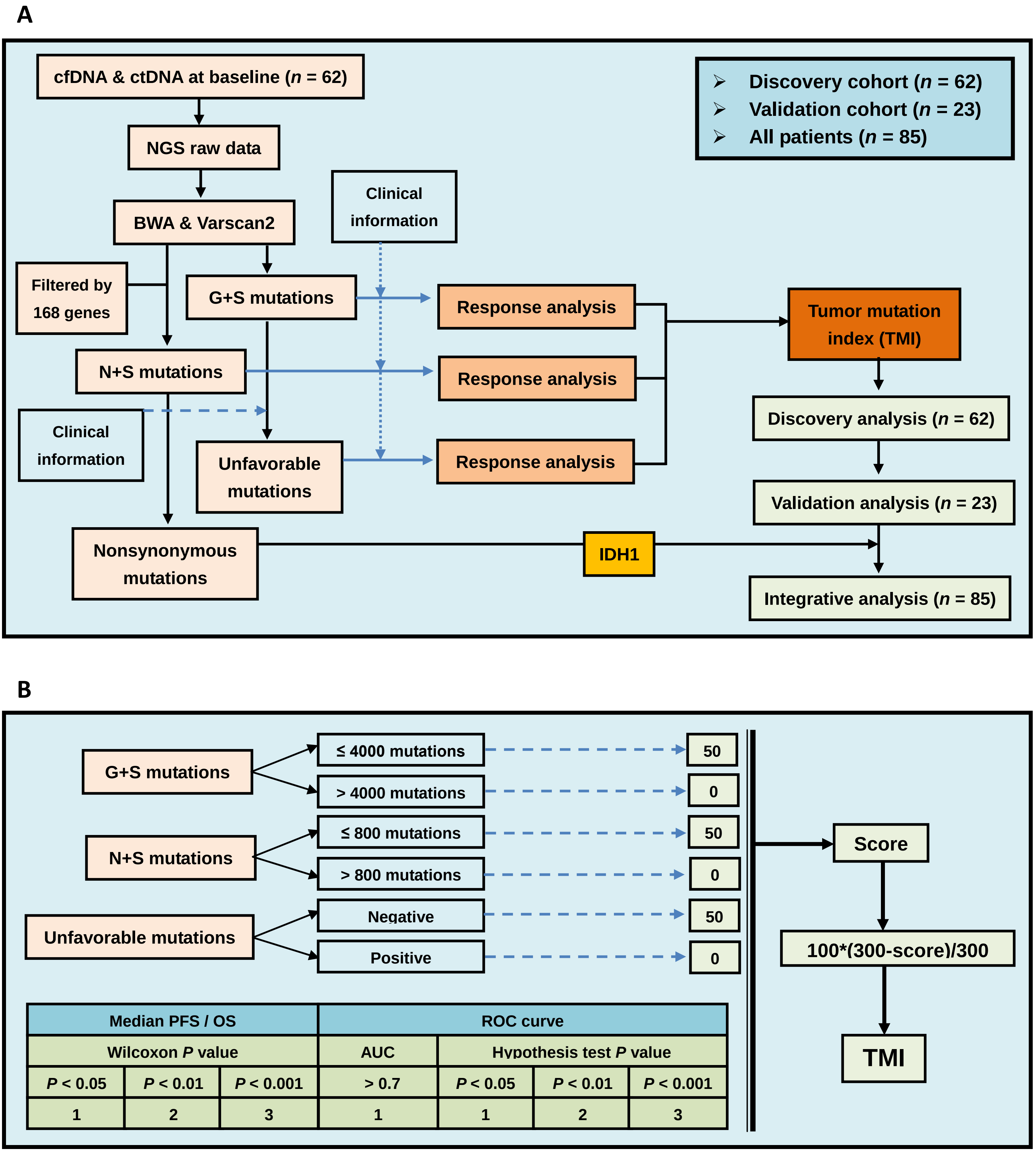
A
cfDNA & ctDNA at baseline (n = 62)
NGS raw data
BWA & Varscan2
Clinical information
Filtered by 168 genes
G+S mutations
N+S mutations
Clinical information
Unfavorable mutations
Nonsynonymous mutations
Response analysis
Response analysis
Response analysis
Discovery cohort (n = 62)
Validation cohort (n = 23)
All patients (n = 85)
Tumor mutation index (TMI)
Discovery analysis (n = 62)
Validation analysis (n = 23)
IDH1
Integrative analysis (n = 85)
B
G+S mutations
≤ 4000 mutations
> 4000 mutations
N+S mutations
≤ 800 mutations
> 800 mutations
Unfavorable mutations
Negative
Positive
50
0
50
0
50
0
Score
100*(300-score)/300
TMI
Median PFS / OS
Wilcoxon P value
P < 0.05
P < 0.01
P < 0.001
1
2
3
ROC curve
AUC
> 0.7
1
Hypothesis test P value
P < 0.05
P < 0.01
P < 0.001
1
2
3

Supplementary Figure 4. (A) Flow diagram for TMI generation and anlotinib response analysis. Total mutations were called from cfDNA and ctDNA. Germline and somatic mutations, nonsynonymous and synonymous mutations, nonsynonymous mutations and unfavorable mutations were used as independent predictor for anlotinib responsive stratification. By integrating the merits and defects of the predictors (G+S MB, N+S MB, and UMS), TMI was generated. *IDH1* mutation is an unfavorable mutation screened from nonsynonymous mutations. TMI plus *IDH1* mutation status was used as predictor for anlotinib response prediction lastly. This analytical model derived from discovery cohort (n = 62 patients), and validated in an independent cohort (n = 23 patients). **(B) Scoring scheme of TMI.** Each patient TMI score was determined by different response analysis of G+S MB, N+S MB, and UMS, respectively. Under different predictors derive from discovery cohort, the patients grouped in anlotinib responders obtained 50 scores and those in anlotinib non-responders scored 0 as BL. For significance scoring scheme, the significant difference wilcoxon P value < 0.05 scored 1, P value < 0.01 scored 2, and P value < 0.001 scored 3, in Kaplan-Meier curve analysis for PFS and OS. The AUC > 0.7 scored 1, and the null hypothesis test P value < 0.05 scored 1, < 0.01 scored 2, and < 0.001 scored 3, in ROC curve analysis for sensitivity and specificity. Total score for each patient is calculated by above factors derived from subgroup analysis under three different regimens. TMI score is obtained by normalization calculation of the total score.

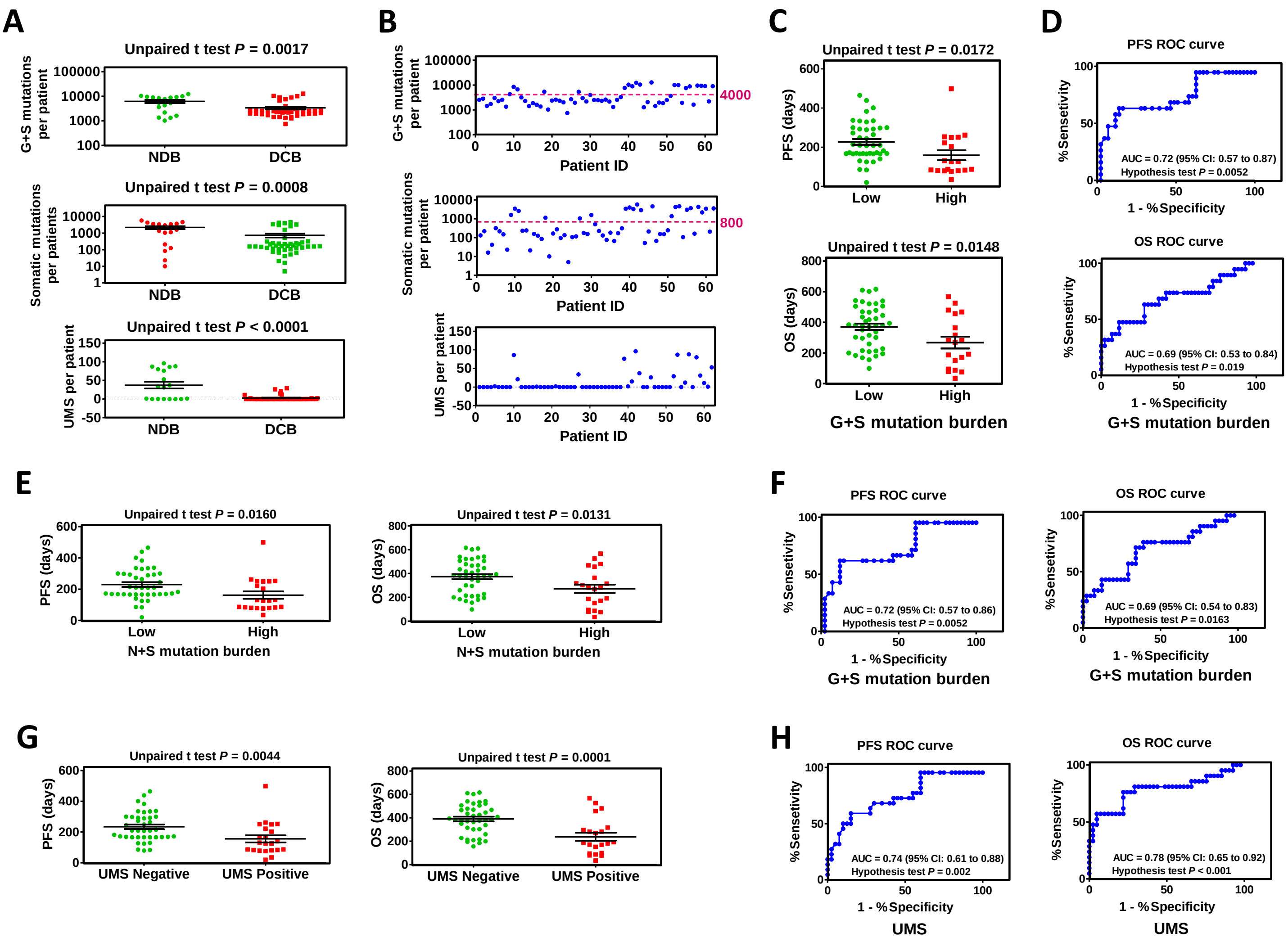

Supplementary Figure 5. G+S mutations, N+S mutations, and UMS used for anlotinib response stratification in discovery cohort, respectively. (A) G+S MB, N+S MB and UMS in plasma circulating DNA from NSCLC patients at BL with DCB (n = 44) and NDB (n = 18). (B) Distribution of G+S MB, N+S MB, and UMS for each patient in discovery cohort. Cutoff = 4000 for G+S MB, cutoff = 800 for N+S MB, negative or positive for UMS, respectively. (C) Absolute PFS and OS analysis between high G+S MB (n = 19) and low G+S MB (n = 43) (PFS: Unpaired t test P = 0.0172; OS: Unpaired t test P = 0.0148). (D) ROC curves for the correlation of G+S MB with anlotinib response. AUC of PFS response prediction is 0.72 (95% CI 0.57 to 0.87, null hypothesis test P = 0.0052) and AUC of OS response prediction is 0.69 (95% CI 0.53 to 0.84, null hypothesis test P = 0.0019). Cutoff = 4000 determined by

ward method. (E) Absolute PFS and OS analysis between high N+S MB (*n* = 19) and low N+S MB (*n* = 43) (PFS: Unpaired t test *P* = 0.0180; OS: Unpaired t test *P* = 0.0131). (F) ROC curves for the correlation of N+S MB with anlotinib response. AUC of PFS response prediction is 0.72 (95% CI 0.57 to 0.86, null hypothesis test *P* = 0.0052) and AUC of OS response prediction is 0.69 (95% CI 0.54 to 0.83, null hypothesis test *P* = 0.0163). Cutoff = 800 determined by ward method. (G) Absolute PFS and OS analysis in the patients with or without unfavorable mutation. UMS is determined by unfavorable mutation numbers. (H) ROC curves for the correlation of UMS with anlotinib response. AUC of PFS response prediction is 0.72 (95% CI 0.57 to 0.86, null hypothesis test *P* = 0.0052) and AUC of OS response prediction is 0.69 (95% CI 0.54 to 0.83, null hypothesis test *P* = 0.0163). In (A), (C), (E), and (G), median and interquartile ranges of total mutations are shown, with individual values for each patient shown with dots.

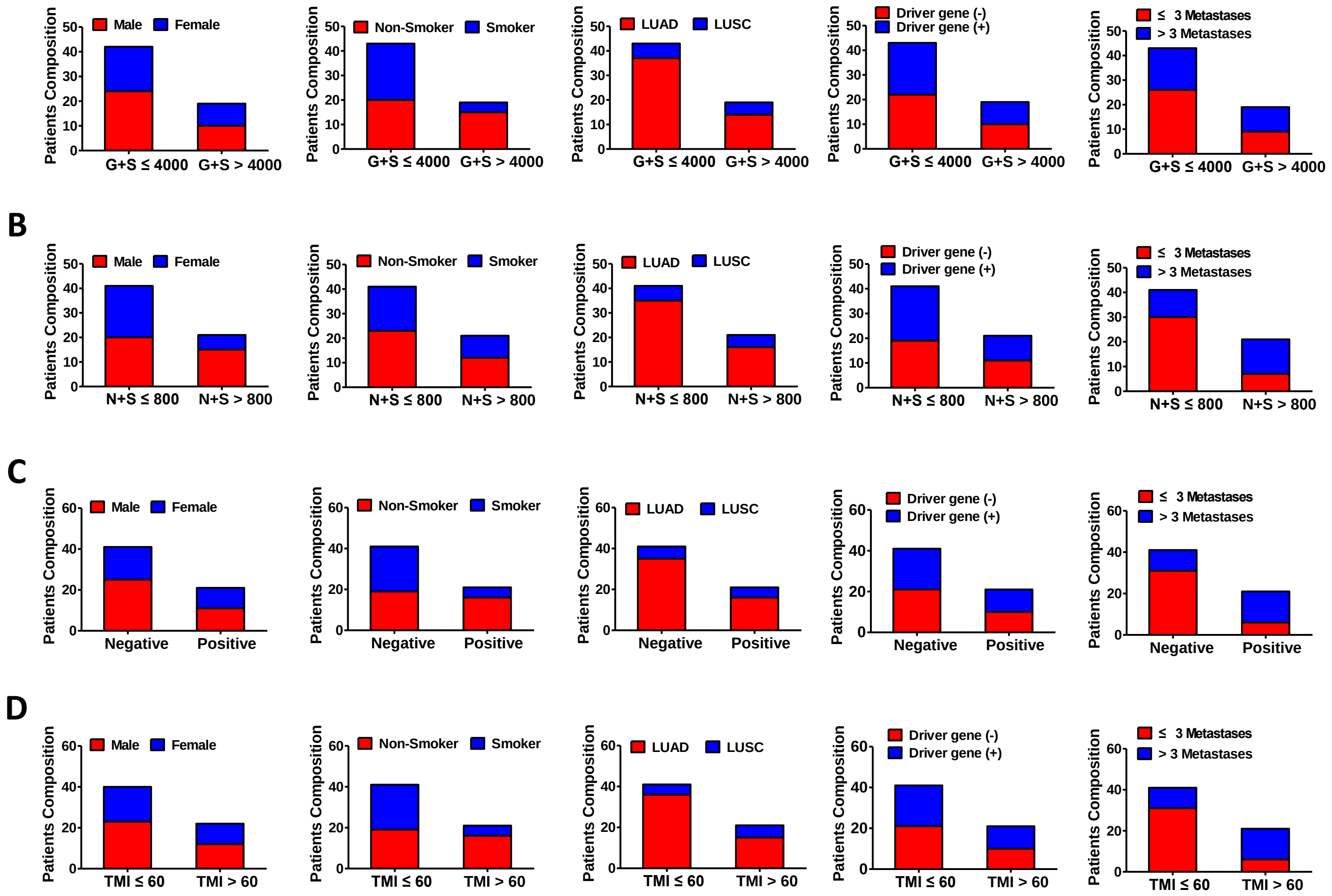

Supplementary Figure 6. Composition analysis in the discovery cohort using different predictors. (A) Distributions of the patients with G+S ≤ 4000 and G+S > 4000 in the subgroups of Male, Female, Non-smoker, Smoker, LUAD, LUSC, Driver gene (+), Driver gene (-), ≤ 3 metastases, and > 3 metastases, respectively. (B) Distributions of the patients with N+S ≤ 800 and N+S > 800 in the subgroups of Male, Female, Non-smoker, Smoker, LUAD, LUSC, Driver gene (+), Driver gene (-), ≤ 3 metastases, and > 3 metastases, respectively. (C) Distributions of the patients with UMS negative and UMS positive in the subgroups of Male, Female, Non-smoker, Smoker, LUAD, LUSC, Driver gene (+), Driver gene (-), ≤ 3 metastases, and > 3 metastases, respectively. (D) Distributions of the patients with TMI ≤ 60 and TMI > 60 in the subgroups of Male, Female, Non-smoker, Smoker, LUAD, LUSC, Driver gene (+), Driver gene (-), ≤ 3 metastases, and > 3 metastases, respectively.

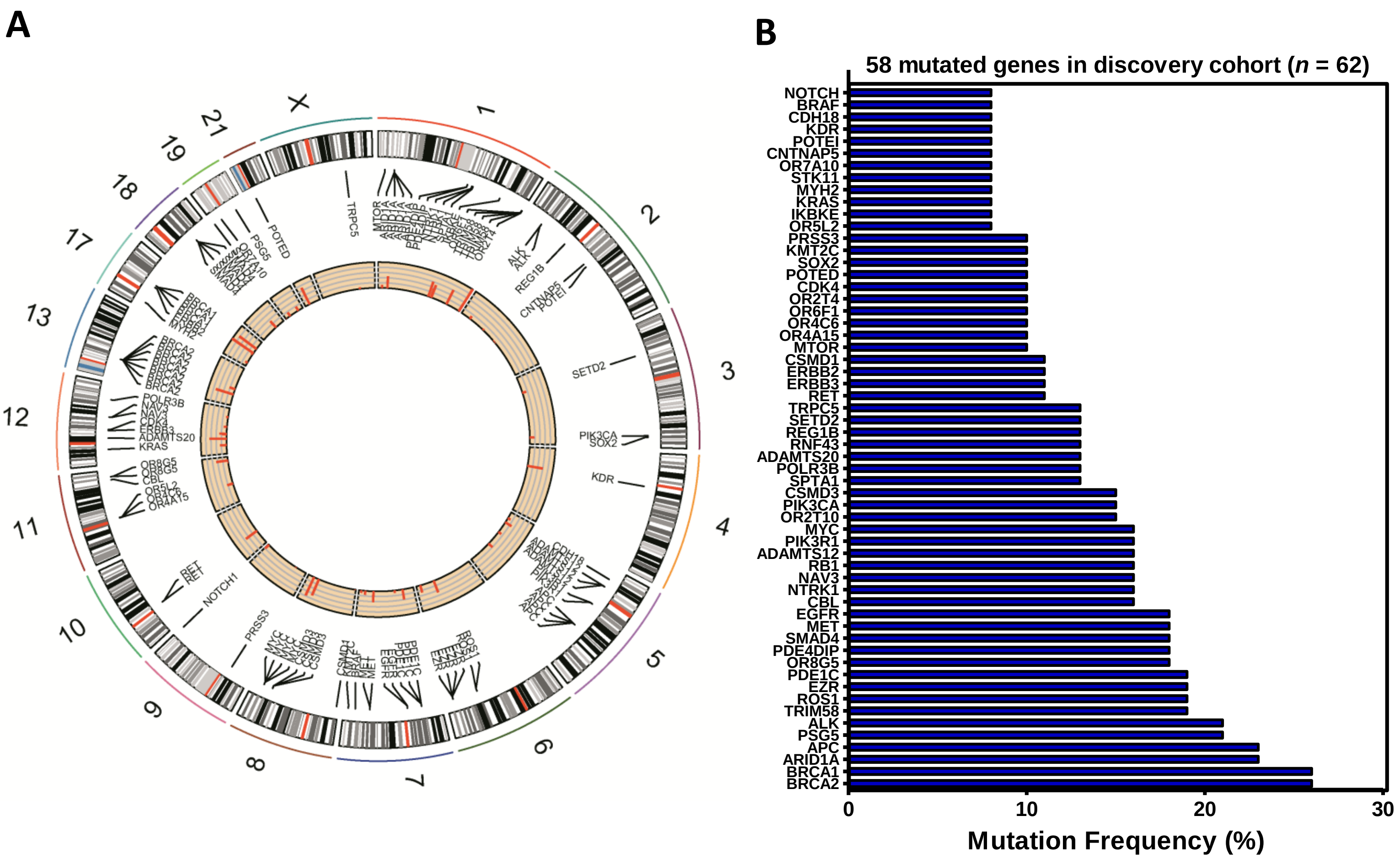

Supplementary Figure 7. (A) The outer circle shows 120 unfavorable mutation points consisted in 58 genes derived from 62 refractory advanced NSCLC patients. The red column in inner circle represents the $Log_{10}^{(P\ value)}$. The Wilcoxon *P* value indicates different response significance to anlotinib in mutation point positive cohort or negative cohort. The *P* value ranges between 1.7E-09 to 1.2E-03. (B) Mutation frequency of 58 unfavorable genes in 62 advanced NSCLC patients cohort.

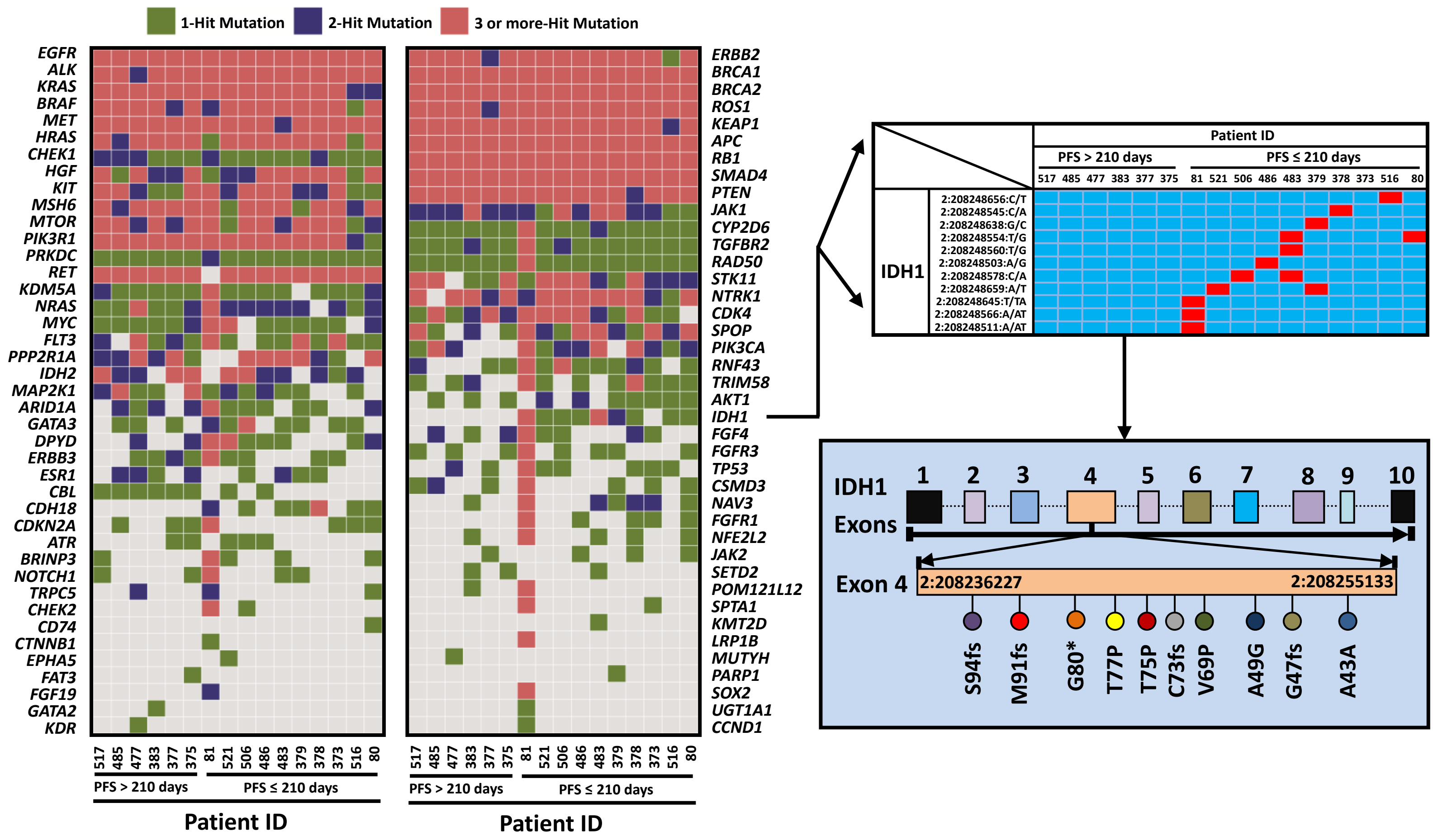

Supplementary Figure 8. ***IDH1*** **exon 4 mutation status determines anlotinib response in full TMI scored patient cohort.** A total of 16 patients with full TMI score. One patient had much more mutations than the other 15 patients. Among the all patients, *IDH1* mutation status in the patients with PFS > 210 days (n = 6) was significantly different from that in the patients with PFS ≤ 210 days (n = 10). These mutations are high effect nonsynonymous mutations and insertion mutations, and all exist in *IDH1* exon 4.

Supplementary Table 1. Clinicopatholigical characteristics of anlotinib DCB patients and anlotinib NB patients.

	**Anlotinib DCB patients (*n*=42) (%)**	**Anlotinib NB patients (*n*=26) (%)**	**P value**
Age, y, (range)	55 (21, 75)	55 (39, 68)	0.9023*
Sex			0.2178#
Female	22 (52)	12 (46)	
Male	20 (48)	14 (54)	
Smoking history			0.3030#
Yes	14 (33)	10 (38)	
No	28 (67)	16 (62)	
Histology			0.0034#
LUAD	36 (86)	19 (73)	
LUSC	6 (14)	7 (27)	
Driver gene status			0.0025#
Positive	19 (45)	8 (31)	
Negative	23 (55)	18 (69)	
Number of metastasis			< 0.0001#
> 3	10 (24)	14 (54)	
≤ 3	32 (76)	12 (46)	

Abbreviations:

*: Mann-Whitney U test; #: Chi-square test

DCB: durable clinical benefit; NB: no benefit; LUAD: lung adenocarcinoma; LUSC: lung squamous carcinoma.

Supplementary Table 2.

Demographic data of 62 refractory advanced NSCLC patients in discovery cohort.

**Characteristic**	**n**	**%**
**Age (Median, Range)**	55(21-71)	
**Gender**		
Male	35	56%
Female	27	44%
**Smoking**		
Non-smoker	38	61%
Smoker	24	39%
**Pathology**		
LUAD	51	82%
LUSC	11	18%
**Anatomic stage**		
IIIB	3	5%
IV	59	95%
**Driver gene status**		
EGFR (19 Del & L858R)	24	39%
EGFR (T790M)	5	8%
ALK	2	3%
Negative	31	50%
**Number of metastases**		
≤ 3	37	60%
> 3	25	40%

Abbreviations:

LUAD: lung adenocarcinoma; LUSC: lung squamous carcinoma

Supplementary Table 3.

Subgroups response analysis using the biomarkers of G+S MB, N+S MB, UMS, and TMI in discovery cohort.

		Median PFS (days)			ROC curve		Median OS (days)			ROC curve	
		G+S < 4000	G+S > 4000	*P* value	AUC	*P* value	G+S < 4000	G+S > 4000	*P* value	AUC	*P* value
**G+S MB**	**Male**	249	84	0.0041	0.7940	0.0073	417	172	0.0050	0.7000	0.0679
	**Female**	203	177	0.5409	0.5864	0.4715	505	282	0.2293	0.6358	0.2579
	**Non-Smoker**	203	172	0.2170	0.6571	0.1123	505	317	0.1293	0.6095	0.2683
	**Smoker**	244	83	0.0002	0.9091	0.0105	417	83	<0.0001	0.9886	0.0023
	**LUAD**	209	131	0.0599	0.6979	0.0305	Undefined	282	0.0078	0.6931	0.0908
	**LUSC**	244	87	0.1527	0.7333	0.2013	382	87	0.2460	0.6667	0.3614
	**Driver gene (-)**	215	126	0.0027	0.8023	0.0061	382	192	0.0077	0.7500	0.0232
	**Driver gene (+)**	209	203	0.2798	0.6616	0.2638	Undefined	317	0.1011	0.6010	0.3841
	**≤ 3 Metastases**	264	127	0.0079	0.8226	0.0135	Undefined	369.5	0.0419	0.6290	0.3228
	**> 3 Metastases**	168	126	0.5878	0.5641	0.5865	214	187	0.3942	0.5962	0.4146
		N+S < 800	N+S > 800	*P* value	AUC	*P* value	N+S < 800	N+S > 800	*P* value	AUC	*P* value
**N+S MB**	**Male**	264	87	0.0055	0.7772	0.0079	Undefined	248	0.0032	0.7065	0.0477
	**Female**	203	177	0.5409	0.5864	0.4715	468	282	0.1400	0.6842	0.1371
	**Non-Smoker**	203	172	0.2170	0.6571	0.1123	505	317	0.1293	0.6095	0.2683
	**Smoker**	298	130	0.0101	0.8250	0.0177	Undefined	120	0.0001	0.8750	0.0062
	**LUAD**	209	131	0.0506	0.6955	0.0263	Undefined	282	0.0057	0.6946	0.0270
	**LUSC**	244	87	0.1527	0.7333	0.2013	382	87	0.2460	0.6667	0.3614
	**Driver gene (-)**	215	126	0.0027	0.8023	0.0061	382	192	0.0077	0.7500	0.0232
	**Driver gene (+)**	208	203	0.4174	0.6286	0.2539	Undefined	310	0.0692	0.6190	0.2908
	**≤ 3 Metastases**	264	130	0.0011	0.8476	0.0047	Undefined	315	0.0155	0.6571	0.2007
	**> 3 Metastases**	167	129	0.9133	0.5130	0.9128	213	235	0.5462	0.5649	0.5841
		UMS (-)	UMS (+)	*P* value	AUC	*P* value	UMS (-)	UMS (+)	*P* value	AUC	*P* value
**UMS**	**Male**	249	87	0.0024	0.8030	0.0045	Undefined	152	0.0004	0.7576	0.0157
	**Female**	185	182	0.3924	0.6118	0.3401	505	235	0.0068	0.7941	0.0121
	**Non-Smoker**	177	168	0.3534	0.6266	0.1973	505	282	0.0039	0.7406	0.0143
	**Smoker**	249	79	<0.0001	0.9619	0.0016	417	87	<0.0001	1.0000	0.0006
	**LUAD**	210	167	0.0637	0.6884	0.0323	Undefined	237	0.0002	0.7625	0.0029
	**LUSC**	244	79	0.0214	0.8667	0.0447	417	87	0.0208	0.8667	0.0447
	**Driver gene (-)**	215	106	0.0032	0.7976	0.0083	378	170	0.0003	0.8571	0.0015
	**Driver gene (+)**	210	168	0.1366	0.6886	0.0867	Undefined	282	0.0023	0.7227	0.0431
	**≤ 3 Metastases**	264	168	0.0392	0.7823	0.0306	Undefined	289	0.0042	0.7204	0.0912
	**> 3 Metastases**	175	126	0.3990	0.6033	0.3899	302	171	0.0266	0.7667	0.0266
		TMI ≤ 60	TMI > 60	*P* value	AUC	*P* value	TMI ≤ 60	TMI > 60	*P* value	AUC	*P* value
**TMI**	**Male**	249	84	0.0003	0.8428	0.0013	Undefined	152	0.0009	0.7386	0.0252
	**Female**	215	167	0.0245	0.6534	0.1828	505	282	0.0028	0.7500	0.0299
	**Non-Smoker**	177	167	0.5160	0.6656	0.0916	Undefined	282	0.0473	0.6500	0.1265
	**Smoker**	249	79	<0.0001	0.9619	0.0016	87	417	<0.0001	1.0000	0.0006
	**LUAD**	210	167	0.0482	0.7009	0.0249	Undefined	282	0.0019	0.7204	0.0139
	**LUSC**	329	83	0.0171	0.9000	0.0285	417	93	0.1323	0.7333	0.2013
	**Driver gene (-)**	215	106	0.0048	0.7881	0.0106	382	181	0.0055	0.7667	0.0180
	**Driver gene (+)**	210	167	0.0449	0.7364	0.0318	Undefined	282	0.0041	0.7091	0.0576
	**≤ 3 Metastases**	264	127	0.0079	0.8226	0.0135	407	370	0.2828	0.6290	0.3228
	**> 3 Metastases**	175	126	0.2544	0.6400	0.2441	258	180	0.1301	0.6800	0.1343

G+S MB: Germline and somatic mutation burden; N+S MB: Nonsynonymous and synonymous mutation burden; UMS: Unfavorable mutation score; TMI: Tumor mutation index; LUAD: lung adenocarcinoma; LUSC: lung squamous carcinoma.
